# Energy concentration and phosphorus digestibility in meat meal, fish meal, and soybean meal fed to pigs

**DOI:** 10.5713/ab.21.0102

**Published:** 2021-06-23

**Authors:** Changsu Kong, Kyoung Hoon Kim, Sang Yun Ji, Beob Gyun Kim

**Affiliations:** 1 Department of Animal Science, Kyungpook National University, Sangju 37224, Korea; 2 Department of International Agricultural Technology, Graduate School of International Agricultural Technology, Seoul National University, Pyeongchang 25354, Korea; 3 Department of Ecofriendly Livestock Science, Institute of Green Bio Science and Technology, Seoul National University, Pyeongchang 25354, Korea; 4 Animal Nutritional Physiology Team, National Institute of Animal Science, Rural Development Administration, Wanju 55363, Korea; 5 Department of Animal Science and Technology, Konkuk University, Seoul 05029, Korea

**Keywords:** Digestible Energy, Meat Meal, Metabolizable Energy, Phosphorus Digestibility, Swine

## Abstract

**Objective:**

The objectives of the present study were to determine digestible energy (DE), metabolizable energy (ME), and standardized total tract digestibility (STTD) of phosphorus (P) in meat meal (MM) and to compare these values with those in fish meal (FM), and soybean meal (SBM) fed to pigs.

**Methods:**

Two experiments were conducted to determine energy concentrations and STTD of P in MM, FM, and SBM fed to growing pigs. In Exp. 1, DE and ME in the 3 test ingredients were measured using 24 barrows with initial body weight (BW) of 77.7±8.3 kg. A corn-based diet and 3 diets containing corn and 22% to 30% of each test ingredient were prepared. In Exp. 2, the STTD of P in the 3 test ingredients was measured using 24 barrows (90.9±6.6 kg BW). Three diets were formulated to contain each test ingredient as the sole source of P.

**Results:**

In Exp. 1, the DE and ME values in MM (3,310 and 2,856 kcal/kg dry matter [DM]) were less (p<0.05) than those in FM (4,121 and 3,572 kcal/kg DM) and SBM (4,390 and 4,032 kcal/kg DM). In Exp. 2, FM (64.3%) had greater (p<0.05) STTD of P than SBM (44.8%) with MM (55.8%) having intermediate STTD of P.

**Conclusion:**

The MM contains less energy concentrations compared with FM and SBM, and digestibility of phosphorus in MM does not differ from that in FM and SBM.

## INTRODUCTION

Dietary energy is critical for the maintenance and growth of pigs [1]. An accurate determination of energy utilization of feed ingredients should precede diet formulations. Soybean meal (SBM), a by-product of oil production from soybeans, is a popular protein source in swine diets [2]. However, due to the increasing price of SBM, feed producers are seeking alternative ingredients to reduce feed cost which usually accounts for over 60% of pig production cost. Meat meal (MM) is defined as “a rendered product from mammal tissues, exclusive of blood, hair, hoof, horn, hide trimmings, manure, stomach and rumen contents” [3]. As MM is a good source of energy as well as protein, this ingredient is often used in swine diets [4]. While amino acid digestibility values in MM have been reported [5–7], the information on the metabolizable energy (ME) contents in MM is very limited, and thus, a calculated ME value in MM is provided in the NRC [1].

Phosphorus (P) is an essential element in pig diets. Animal protein sources generally contain a relatively large quantity of P. However, the biological availability of P in feed ingredients varies [1,8–10]. Standardized total tract digestible (STTD) P has been suggested as a measurement for biologically available P in swine diets [1]. Information on the STTD of P in MM, to our knowledge, is not available. Therefore, the objectives of the present study were to determine digestible energy (DE), ME, and STTD of P in MM and to compare these values with those in fish meal (FM), and SBM fed to pigs.

## MATERIALS AND METHODS

All the experimental procedures for both experiments were approved by the Institutional Animal Care and Use Committee at Konkuk University (KU11008).

### Exp. 1. Digestible and metabolizable energy contents

#### Animals and experimental diets

Twenty-four barrows with an initial body weight (BW) of 77.7±8.3 kg were used to determine DE and ME in MM, FM and SBM (Table 1). The animals were individually housed in metabolism crates equipped with a feeder and a nipple drinker. The animals were assigned to 4 diets with 6 pigs per diet in a randomized complete block design.

Four experimental diets consisted of a reference diet and 3 test diets (Table 2). In the reference diet, corn was used as the sole source of energy. In the test diets, MM, FM, or SBM was added at 23%, 22%, or 30% of diet, respectively, to partly replace corn in the reference diet. Vitamins and trace minerals were included in all diets to meet or exceed nutrient requirement estimates [8].

#### Feeding and sample collection

The daily feed allowance was calculated as 2.7% of initial BW and divided into 2 equal meals. The feed allowance was approximately 2.5 times the estimated maintenance requirement for ME (197 kcal/kg BW^0.60^) [1]. Water was freely accessible throughout the experiment. The study consisted of a 5-d adaptation period followed by a 5-d period of total but separate collection of feces and urine. Fecal collection initiated and ended with the appearance of chromic oxide-marked feces as described by Kong and Adeola [11]. Urine collection started at 1400 h on d 6 and ended at 1400 h on d 11.

#### Chemical analyses

The total quantities of collected feces and urine were stored at −20°C immediately after collection. At the completion of the experiment, the frozen feces were dried in a freeze drier, and ingredient, diet, and fecal samples were finely ground prior to chemical analyses. Ingredient samples were analyzed in duplicate for ash (method 942.05), and ether extract (method 920.39) [12]. Nitrogen in ingredient samples for calculation of crude protein (CP) content was analyzed in duplicate by the Kjeldahl method (Kjeltec 1035; Foss, Hillerod, Denmark; method 976.05) [12]. Dry matter (DM) analysis was also performed in duplicate for ingredients, diets, and feces (method 930.15) [12]. All samples of ingredients, diets, feces, and urines were measured for gross energy (GE) [2] using an adiabatic bomb calorimeter (Model C2000, IKA, Staufen, Germany).

#### Calculations and statistical analyses

The DE or ME contents in the experimental diets were calculated by subtracting the GE of feces or both feces and urine GE from the GE of an experimental diet, respectively. The DE and ME of each ingredient were then calculated using the difference procedure as described by Kong and Adeola [11].

The experimental data were analyzed using MIXED proce dures of SAS (SAS Inst. Inc., Cary, NC, USA). The independent variables in the initial model included diet as a fixed variable and block as a random variable, but block was excluded from the model because the block effect was not significant. Least squares means were calculated, and the means were separated using the PDIFF option with Tukey’s adjustment. The pig was the experimental unit and an alpha level of 0.05 was used to determine significance.

### Exp. 2. Phosphorus digestibility

#### Animals and experimental diets

Twenty-four barrows with an initial BW of 90.9±6.6 kg were used to determine P digestibility in MM, FM, and SBM. The pigs were individually housed in the metabolism crates. The 24 animals were assigned to 4 diets with 6 pigs per diet in a randomized complete block design. Three experimental diets were formulated to contain MM, FM, and SBM as the sole source of P (Table 3). Additionally, a P-free diet was also prepared to estimate the basal endogenous losses (BEL) of P [9,13]. All diets were supplemented with adequate amounts of vitamins and minerals except P according to the requirement estimates suggested by the NRC [8].

#### Feeding and sample collection

The daily feed allowance was calculated as 2.7% of initial BW and divided into 2 equal meals. The feed allowance was approximately 3.1 times the estimated maintenance requirement for ME (197 kcal/kg BW^0.60^) [1]. Water was freely accessible throughout the experiment. The study consisted of a 5-d adaptation period followed by a 5-d period of total collection of feces. Fecal collection initiated and ended with the appearance of chromic oxide-marked feces as described by Kong and Adeola [11].

#### Chemical analyses

The total quantity of collected feces was stored at −20°C immediately after collection. At the completion of the experiment, the frozen fecal samples were dried in a freeze drier, and diet and fecal samples were finely ground prior to chemical analyses. The DM analysis was also performed in duplicate for diets and feces (method 930.15) [12]. The P concentrations in the ingredients, diets and fecal samples were determined using a spectrophotometer (method 946.06) [12].

#### Calculations and statistical analyses

Apparent total tract digestibility (ATTD) of P was calculated using the following equation [9,13]:

ATTD of P (%)=[(Pi-Po)/Pi]×100

where, Pi and Po represent total P intake (g) and total fecal P output (g), respectively, during 5-d collection period. The BEL of P were estimated from pigs fed the P-free diet using the following equation [9,13]:

BEL of P (mg/kg DM intake)=[(Po/DMi)×1,000]

where, DMi represents DM intake (kg) during the 5-d collection period. The STTD of P in each diet was calculated by correcting the ATTD of P for BEL of P using the following equation [9,13]:

STTD of P (%)=ATTD of P+(BEL of P×DMi)/(Pi×1,000)×100%

Statistical analysis procedures for the data from Exp. 2 was performed as described for Exp. 1.

## RESULTS

### Exp. 1. Digestible and metabolizable energy contents

All pigs consumed their diets well, and feces and urine collection procedures were successful in all pigs, resulting in similar diet intake in all experimental diets (Table 4). There was no difference in GE intake and urine GE output among pigs fed experimental diets. Fecal GE output was greater (p< 0.05) for pigs fed the MM diet than for pigs fed all other diets except the FM diet. The ATTD of energy in MM diet was less (p<0.05) than in the corn and SBM diets but was not different from that in the FM diet. The DE in the MM diet was less (p<0.05) than that in the SBM and FM diets.

The DE and ME values in MM were less (p <0.05) than those in SBM and FM (Table 5). The DE in corn was less than that in FM and SBM on an as-fed basis. However, the ME in corn was not different from that in FM and SBM.

### Exp. 2. Phosphorus digestibility

All pigs consumed their diets well, and feces collection procedures were successful in all pigs. The amounts of feed consumption were comparable in the 3 diet groups (Table 6). Phosphorus intake was greater (p<0.05) for pigs fed the MM and FM diets compared with pigs fed the SBM diet. The concentrations of P in feces from pigs fed the MM or FM diets were greater (p<0.05) than that from pigs fed the SBM diet. The FM had greater (p<0.05) ATTD and STTD of P than SBM, with MM having intermediate ATTD and STTD of P.

## DISCUSSION

Animal protein sources are often used in swine diets as an amino acid source. However, energy and P concentrations in the animal protein sources are also high [1]. Therefore, energy values and P digestibility of animal protein sources are critical for precise swine diet formulations. As MM is potentially a good source of energy and P, the energy values and P digestibility were determined in the present work.

In the statistical analysis procedures for both experiments, the diet effect was the sole independent variable in the statistical model, but the block effect was not included in the model. Although a randomized complete block design was used to achieve similar mean BW among the dietary groups, the variation of BW within a dietary group was not that large, which likely resulted in no effects of block on energy and P digestibility. This observation is in agreement with Kim et al [14] who reported no effects of BW within a relatively narrow range on DM digestibility.

The GE concentrations of corn, FM, and SBM used in the present study were reasonably close to the values in the literature [1,9,10]. The concentrations of P and Ca in the MM used in the present study met the criterion provided by the AAFCO [3] but the concentration of CP was greater compared with the value reported in the NRC [1], which is probably attributed to the different source of MM in the present work [15]. The GE in the MM in the present work was a bit less (4,400 vs 4,497 kcal/kg) than the value in the NRC [1] most likely due to less ether extract (8.5% vs 11.1%) and greater CP (64.5% vs 56.4%) contents. The concentration of nutrients in the SBM used in the present study was comparable to the reference values for SBM with similar CP content [1,5,9,16,17].

The difference procedure was employed to calculate DE and ME values for test ingredients and corn was used as the basal ingredient. Due to the nature of the difference procedure, an accurate determination of energy values in corn is an essential prerequisite for an accurate evaluation of test ingredients [9]. In the present work, the concentrations of DE and ME in the corn agreed with previously published values [1,8,9].

The DE and ME values (4,390 and 4,032 kcal/kg DM, re spectively) calculated for SBM in the present study were slightly greater than the values of 4,022 and 3,661 kcal/kg DM reported by the NRC [1]. and values of 4,000 and 3,646 kcal/kg DM from Sauvant et al [17]. But the DE and ME contents in SBM used in the present study were comparable to the values reported by Kim et al [9].

As GE in the MM in the present work was similar to the values in the NRC [1] and Sauvant et al [17], the relatively low DE and ME values in MM in the present work is due to low energy digestibility. The factors potentially affecting energy digestibility include nutrient compositions, feed intake, and BW of pigs [18–20]. However, the specific reason for the relatively high DE and ME in MM in the present work is unknown.

The DE content (4,121 kcal/kg DM) for FM used in the present study was slightly less than published values of 4,224 and 4,544 kcal/kg in the NRC [1] and Kim et al [9], respectively, but greater than value of 3,952 kcal/kg reported by Sauvant et al [17]. The ME content in FM was also slightly less than values reported in the NRC [1] and Kim et al [9], however the ME value was comparable to the ME reported by Sauvant et al [17]. The reason for these differences between the present data and published values is most likely due to differences in various sources of fish or fishery by-products used to produce FM and different manufacturing procedures for FM [21]. Additionally, the growth stage of the pigs may also have influenced the energy utilization rate of the FM. Generally, nursery pigs have a bit less energy digestibility compared with grow-finishing pigs [17].

The lower DE values in MM compared with FM and SBM is likely due to the low energy digestibility in MM. While the GE concentration in MM was relatively comparable to the values in FM and SBM, the DE:GE in MM (0.719) was quite less than those in FM (0.854) and SBM (0.916). The inclusion rate of a test ingredient and the energy digestibility of experimental diets are reflected in the DE:GE of a test ingredient. The test ingredients were included at 22% to 30% in the experimental diets and the energy digestibility was the lowest in the MM diet. The low energy digestibility of MM is possibly due to the low CP digestibility in MM. In our previous experiment employing the same MM and SBM [5] as in the present work, standardized ileal digestibility of CP in MM was less (63.5% vs 88.8%; p<0.001) than that in SBM. The amino acids in MM may have become unavailable during the rendering process possibly by overheating [22–24].

The lower ME:DE in MM (0.863) and FM (0.867) com pared with that in SBM (0.918) is reasonable as MM and FM contain greater CP contents compared with SBM. The dietary CP contents are well known to be negatively correlated with ME:DE [25,26]. The energy values reported in the present work are important information for accurate diet formulations. However, the variability of MM sources should be considered as the energy digestibility and nutrient contents in MM sources can vary [27] likely depending on the production process and the raw materials.

The greater daily P intake in the pigs fed the MM and FM diets compared with the SBM group is mainly due to the high P concentrations in the MM and FM as the amounts of daily feed intake were quite similar among the 3 diet groups.

Animal-derived ingredients contain less phytate P:total P compared with plant-derived ingredients, and phytate P is well known to be less digestible compared with non-phytate P [1] The greater ATTD and STTD of P in MM and FM than in SBM agree with She et al [28]. The STTD of P for SBM in the present work is reasonably close (45% vs 48%) to the value in the NRC [1]. However, the STTD of P for FM in the present work was less (64% vs 82%) than that in the NRC [1]. The variability among FM sources may have influenced P digestibility, but the clear reason for this discrepancy is unknown.

The BEL of P were calculated as 182 mg per kg DM intake in the present work. This value is within the range of values in the literature [9,10,13,29] and is close to 190 mg per kg DM intake that was suggested by the NRC [1]. The BEL of P have been reported to little variation among the experiments. Researchers may calculate STTD of P using 190 mg per kg DM intake when only ATTD of P is available as in the work by Sung et al [30].

## CONCLUSION

The MM contains less ME concentration compared with FM and SBM, and digestibility of P in MM does not differ from that in FM and SBM. Present information may serve as reference values when MM is included in swine diets.

## Figures and Tables

**Table 1:** Energy and nutrient concentrations in corn, meat meal, fish meal, and soybean meal, as-is basis

Item	Corn	Meat meal	Fish meal	Soybean meal
Gross energy (kcal/kg)	3,900	4,400	4,445	4,204
Dry matter (%)	86.6	96.5	92.2	87.7
Crude protein (%)	7.44	64.50	57.69	47.00
Ether extract (%)	3.15	8.54	9.94	1.42
Ash (%)	1.26	21.22	20.10	5.50
Calcium (%)	0.16	7.63	6.09	0.33
Phosphorus (%)	0.26	3.64	2.83	0.64

**Table 2:** Ingredient and nutrient compositions of experimental diets used in Exp. 1, as-fed basis

Items	Corn	Meat meal	Fish meal	Soybean meal
Ingredient composition (%)				
Corn	97.7	76.1	77.1	67.9
Meat meal	-	23.0	-	-
Fish meal	-	-	22.0	-
Soybean meal, 47% crude protein	-	-	-	30.0
Limestone	0.7	-	-	0.6
Dicalcium phosphate	0.7	-	-	0.6
Salt	0.4	0.4	0.4	0.4
Vitamin-trace mineral premix^1)^	0.5	0.5	0.5	0.5
Chemical composition				
Gross energy (kcal/kg)	3,832	3,975	4,020	3,949
Crude protein (%)	8.1	20.1	20.2	19.9
Ether extract (%)	3.81	5.73	5.07	3.55
Calcium (%)	0.50	1.84	1.22	0.52
Available phosphorus (%)	0.17	0.83	0.66	0.19

1) Supplied per kg of complete diet: vitamin A, 11,128 IU; vitamin D_3_, 2,204 IU; vitamin E, 66 IU; vitamin K, 1.42 mg; thiamin, 0.24 mg; riboflavin, 6.58 mg; pyridoxine, 0.24 mg; vitamin B_12_, 0.03 mg; D-pantothenic acid, 23.5 mg; niacin, 44 mg; folic acid, 1.58 mg; biotin, 0.44 mg; Cu, 10 mg as copper sulfate; Fe, 125 mg as iron sulfate; I, 1.26 mg as potassium iodate; Mn, 60 mg as manganese sulfate; Se, 0.3 mg as sodium selenite; and Zn, 100 mg as zinc oxide.

**Table 3:** Ingredient and nutrient composition of experimental diets used in Exp. 2, as-fed basis

Item	Diet			

	Meat meal	Fish meal	Soybean meal	P-free
Ingredient composition (%)				
Corn starch	63.63	62.85	48.20	51.47
Meat meal	12.00	-	-	-
Fish meal	-	12.00	-	-
Soybean meal, 47% crude protein	-	-	35.00	-
Sucrose	12.00	15.00	12.00	20.00
Soybean oil	3.60	2.70	2.80	3.90
Gelatin	7.00	6.00	-	19.00
Cellulose	-	-	-	3.00
DL-Methionine	0.15	-	-	0.20
L-Threonine	0.07	-	-	-
L-Tryptophan	0.10	0.05	-	0.10
L-Histidine	-	-	-	0.05
L-Isoleucine	0.05	-	-	0.03
Limestone	-	-	0.60	0.85
Potassium carbonate	0.40	0.40	0.40	0.40
Magnesium oxide	0.10	0.10	0.10	0.10
Salt	0.40	0.40	0.40	0.40
Vitamin-trace mineral premix^1)^	0.50	0.50	0.50	0.50
Chemical composition (%)				
Dry matter	89.9	89.2	89.2	90.3
Crude protein	16.1	11.2	16.3	24.8
Ash	3.52	3.24	3.26	1.11
Phosphorus	0.399	0.367	0.214	-

1) Supplied per kg of complete diet: vitamin A, 11,128 IU; vitamin D_3_, 2,204 IU; vitamin E, 66 IU; vitamin K, 1.42 mg; thiamin, 0.24 mg; riboflavin, 6.58 mg; pyridoxine, 0.24 mg; vitamin B_12_, 0.03 mg; D-pantothenic acid, 23.5 mg; niacin, 44 mg; folic acid, 1.58 mg; biotin, 0.44 mg; Cu, 10 mg as copper sulfate; Fe, 125 mg as iron sulfate; I, 1.26 mg as potassium iodate; Mn, 60 mg as manganese sulfate; Se, 0.3 mg as sodium selenite; and Zn, 100 mg as zinc oxide.

**Table 4:** Energy balance in pigs fed experimental diets (as-fed basis),^1)^ Exp. 1

Item^2)^	Diet				SEM	p-value

	Corn	Meat meal	Fish meal	Soybean meal		
Diet intake (kg/d)	2.08	2.07	2.13	2.07	0.099	0.968
Feces output (kg/d)	0.198^b^	0.285^a^	0.272^a^	0.199^b^	0.0100	<0.001
Urine output (kg/d)	4.27	3.30	4.04	5.06	0.129	0.841
GE in feces (kcal/kg)	4,833^a^	4,515^ab^	4,182^b^	4,751^a^	135.4	0.014
GE in urine (kcal/kg)	128.8	165.9	141.5	95.7	15.53	0.043
GE intake (kcal/d)	7,971	8,229	8,579	8,190	387.4	0.740
Fecal GE output (kcal/d)	957^b^	1,287^a^	1,133^ab^	945^b^	51.5	0.001
Urinary GE output (kcal/d)	371	508	551	463	76.5	0.413
ATTD of energy (%)	87.8^a^	84.4^b^	86.7^ab^	88.4^a^	0.66	0.003
Feed DE (kcal/kg)	3,363^b^	3,354^b^	3,490^a^	3,493^a^	25.5	0.001
Feed ME (kcal/kg)	3,174	3,106	3,229	3,267	53.9	0.238

SEM, standard error of the means; GE, gross energy; ATTD, apparent total tract digestibility; DE, digestible energy; ME, metabolizable energy.

1) Each least squares mean represents 6 observations.

2) Diet intake, feces output, and urine output were based on 5 d of collection.

a,b Means within a row without a common superscript letter differ (p<0.05).

**Table 5:** Digestible energy and metabolizable energy for corn, meat meal, fish meal, and soybean meal in pigs^1)^, Exp. 1

Item	Ingredient				SEM	p-value

	Corn	Meat meal	Fish meal	Soybean meal		
Digestible energy (kcal/kg)						
As-fed basis	3,443^b^	3,194^b^	3,798^a^	3,851^a^	64.8	<0.001
Dry matter basis	3,975^b^	3,310^c^	4,121^ab^	4,390^a^	71.0	<0.001
Metabolizable energy (kcal/kg)						
As-fed basis	3,249^ab^	2,756^b^	3,293^a^	3,537^a^	122.5	0.004
Dry matter basis	3,751^a^	2,856^b^	3,572^a^	4,032^a^	134.1	<0.001

SEM, standard error of the means.

1) Each least squares mean represents 6 observations.

a–c Means within a row without a common superscript letter differ (p<0.05).

**Table 6:** Phosphorus digestibility of meat meal, fish meal, and soybean meal in pigs^1)^

Item	Diet			SEM	p-value

	Meat meal	Fish meal	Soybean meal		
Diet DM (%)	89.9	89.2	89.2	-	-
Diet P (%)	0.399	0.367	0.214	-	-
Diet intake (kg/d)	2.82	3.02	2.78	0.294	0.832
DM intake (kg/d)	2.54	2.68	2.48	0.264	0.841
P intake (g/d)	11.3^a^	11.1^a^	6.0^b^	0.93	0.002
Fecal DM (%)	96.7^a^	96.3^a^	93.3^b^	0.40	<0.001
Fecal P (%)	6.23^a^	5.33^a^	3.86^b^	0.246	<0.001
Fecal output (g/d)	87.8	86.2	100.6	16.20	0.787
DM output (g/d)	84.8	83.0	94.2	15.76	0.860
P output (g/d)	5.3	4.6	3.8	0.72	0.403
ATTD of DM (%)	96.6	97.0	96.3	0.41	0.471
ATTD of P (%)	51.7^ab^	59.8^a^	37.2^b^	5.00	<0.001
STTD^2)^ of P (%)	55.8^ab^	64.3^a^	44.8^b^	5.00	<0.001

SEM, standard error of the mean; DM, dry matter; P, phosphorus; ATTD, apparent total tract digestibility; STTD, standardized total tract digestibility.

1) Each least squares mean represents 6 observations.

2) Values for STTD were calculated by correcting ATTD values for the basal endogenous losses of P (182±25.3 mg/kg DM intake) determined in pigs fed the P-free diet.

a,b Means within a row without a common superscript letter differ (p<0.05).
